# Safety and Efficacy Evaluation of Ultrasound Aspirators in Intramedullary Spinal Cord Tumors Surgery: An Experimental Study on a Swine Model

**DOI:** 10.3390/brainsci15070670

**Published:** 2025-06-21

**Authors:** Mauro Palmieri, Alessandro Pesce, Mattia Capobianco, Massimo Corsini, Giorgia Iovannitti, Fulvio Aloj, Giuseppa Zancana, Vincenzo Esposito, Maurizio Salvati, Antonio Santoro, Gianpaolo Cantore, Alessandro Frati

**Affiliations:** 1 Human Neurosciences Department, Neurosurgery Division, “Sapienza” University, 00161 Rome, Italy; mauro.palmieri10@gmail.com (M.P.); mattia.capobianco@uniroma1.it (M.C.); massimo.cosini@uniroma1.it (M.C.); giorgia.iovannitti@uniroma1.it (G.I.); giuseppa.zancana@uniroma1.it (G.Z.); antonio.santoro@uniroma1.it (A.S.); neurochirurgia.umberto1@gmail.com (G.C.); alex.frati@gmail.com (A.F.); 2 Department of Neurosurgery, University of Rome ‘’Tor Vergata”, 00133 Rome, Italy; salvatimaurizio1959@gmail.com; 3 IRCCS “Neuromed”, 86077 Pozzilli, Italy; aloj@neuromed.it (F.A.); vincenzo.esposito@uniroma1.it (V.E.)

**Keywords:** ultrasound aspirator, CNS tumors, spinal cord, intramedullary tumors, animal model

## Abstract

**Introduction**: Intradural extramedullary and intramedullary spinal tumors are rare, complex to treat, and require advanced surgical techniques. Ultrasonic aspirators, commonly used for tumor removal, can cause sensory and motor deficits, including loss of motor evoked potentials (MEPs). This study aims to evaluate the safety and efficacy of ultrasonic aspirators in intramedullary tumor surgery using a swine model, comparing different systems and techniques. **Methods**: Ten pigs underwent D1-D3 laminectomy and myelotomy, with adipose tissue simulating a tumor. The ultrasonic aspirators were tested under varying conditions (fragmentation power, suction, application time, and vibration mode). The primary endpoint is to evaluate the impact of the chosen variables on motor function damage. The secondary endpoints are histological evaluation of the type of damage caused by ultrasound aspirators and the effect of steroid drugs on MEPs’ impairment recovery. **Results**: Ultrasound aspirators can cause a significant MEP signal reduction when used in continuous mode, with fragmentation power >30 for more than 2 min (*p* < 0.001). Suction does not affect MEPs. When used in alternating/pulsatile mode, fragmentation power and application time do not affect MEPs. The two-way ANOVA analysis on the interaction between fragmentation power and application time in continuous mode did not demonstrate a significant interaction (*p* = 0.155). Time alone does not affect motor damage (*p* = 0.873). Betamethasone can restore MEPs’ signal after damage if administered immediately. **Conclusions**: Using ultrasonic aspirators in an animal model of intramedullary tumor surgery is safe. The main factor that resulted in the responsibility of motor function impairment is the fragmentation power.

## 1. Introduction

Extramedullary intradural spinal tumors (EM-SPT) and intramedullary spinal tumors (IM-SCT) are rare and account for 2–4% of CNS tumors [1,2]. Intramedullary tumors represent one of the most complex conditions to treat in neurosurgery, where experience, technical skill, and technology are key components. Modern intramedullary tumor surgery is based on using the latest technology, from the intraoperative microscope to dedicated microsurgical instrumentation, to intraoperative neurophysiological monitoring, and the use of different generation ultrasonic aspirators.

In the experience of our institution, which published one of the largest worldwide series of patients operated on for intramedullary tumors [3,4], we have noted how often during this type of surgery the use of ultrasonic aspirators (particularly useful to achieve tumor debulking without exerting traction on the spinal cord microcirculation) during tumor excision results in a reduction and sometimes disappearance of sensory and motor potentials, particularly the D wave, a predictive index of definitive motor damage in the patient [3,4,5,6]. This affects the attitude of the operating surgeon, influencing the extent of tumor removal.

The mechanism of action of ultrasound is to bring about mechanical damage on the tumor cells that are destroyed [7]; how the ultrasound is generated, the intensity of suction, vibration, and the amount of heat developed are all factors that can affect the functional, anatomical, and vascular integrity of the spinal cord (vasospasm, functional or anatomical damage of the nerve pathways).

Although the possible complications related to the use of ultrasonic aspirators in intramedullary tumor surgery have already been described in some case reports [8,9,10,11,12], to date, there is still a lack of studies giving exact indications regarding the use of this instrument for resection of these lesions.

Because the use of ultrasonic aspirators is particularly useful in the resection of Central Nervous System (CNS) tumors, including intramedullary tumors, the need for accurate scientific evidence regarding the safety/efficacy profile of these valuable surgical tools is relevant.

Such verification can only be performed in a complex animal model and be validated by a careful study that simulates, in size and anatomy, the reference human one.

This study aims to experimentally validate what is consistently observed in surgical practice. Adopting the same surgical procedure applied in human medicine and the same technologies and instruments used in intramedullary tumor surgery, this study aims to compare different ultrasonic aspirator systems and different ways of using these aspirators on an animal model of intramedullary tumor to define the ideal safety–efficacy profile of this tool. The final objective of the experiments is to test the role of ultrasounds as a possible cause.

## 2. Materials and Methods

### 2.1. Animal Model

Our experience excludes any possibility of using anything other than a live animal model, specifically a swine model [13]. Our preliminary experimental investigations on small laboratory animals (Rattus Norvegicus) brought us to consider that the swine spinal cord is far more similar to the human spinal cord regarding its anatomy, physiology, and, most of all, dimension so as to create a high compatibility and reproducibility between the experimental and the real surgical setting for what concerns the specific armamentarium object of the investigation. The swine model proves to be better for the size of the instrumentation used (both for the size of the tips, in ex vivo, of the aspirators and for the detection of potentials) [14]. Smaller instruments may invalidate proper use and do not allow comparison and reproduction of the procedure during surgery in human clinical practice.

In conclusion, sensory and motor monitoring can only be performed on live animals. The swine represents the ideal animal because of the similarity of the anatomy and physiology of the compartment under study, superimposed on that of humans [15]. The size of the subject, a swine of about 50 kg, and the possibility of using the same monitoring and instruments, both surgical and those under study, make it the ideal candidate.

### 2.2. Ethical Statement

The experimental protocol and study design were approved by the Review Board of our institution after careful review of the surgical, clinical, neurophysiological, and radiological methods outlined in the article, with protocol number 0877/2023 and study number 7313, 10 October 2023. This study is fully consistent with the Declaration of Helsinki on Human and Animal Rights in Biomedical Research. Furthermore, the present research was conducted in full respect of the ARRIVE guidelines [16] and complied with the Guide for the Care and Use of Laboratory Animals (Institute of Laboratory Animal Resources, Commission on Life Sciences, National Research Council, Washington: National Academy Press, 1996).

### 2.3. Setting

All experimental surgical procedures featured in this study were performed at the Biotechnology Center, AORN “Cardarelli,” Naples, Italy.

For our set-up, 12 large white adult swines were used with a weight ranging from 50 ± 10 kg. Animals were obtained from the Biotechnology Center AORN “Cardarelli”, Naples, Italy, and received a full health check upon arrival at the animal care facilities by a veterinarian. The swines were housed on a solid floor covered with straw. The animals were fed a full standard diet by animal keepers until the evening before the experiment, with free access to water. A veterinarian specialist experienced in porcine experiments was present throughout the whole procedure.

All experiments were conducted with the aid of cranial and cortical neurophysiological monitoring, using the NIM-ECLIPSE™ Spinal System (Medtronic©, Minneapolis, MN, USA).

All surgical procedures consisted of a D1-D3 laminectomy and D1-D2 myelotomy of about 1 cm with insertion of a small piece of subcutaneous tissue of about 0.5 cm^3^ as detailed below. Ultrasonic aspirators were used at different vibration modes (continuous, alternating, and pulsatile), different suctions, different fragmentation power and selectivity, and for increasing periods from one to five minutes. Ultrasonic aspirators were first tested on an apple that served as a phantom.

### 2.4. Experimental Design

The ultrasonic aspirators had the following settings: the frequency was set at 34 kHz and a standard straight micro tip was used during the experimental phase in all cases. The reason behind the choice of applying the same settings and tips is related to the need to make the use of the two instruments homogeneous and to perform the experiments under the same conditions.

The primary endpoint was the evaluation on a porcine intramedullary tumor model of motor damage in terms of reduction (>50%) or disappearance of the potential assessed by neurophysiological monitoring for the different types of aspirators by evaluating the following parameters, categorized as independent variables: power, application time, suction, and vibration mode.

1. Power was increased progressively as follows: 10-20-30-40-50. Classified as an ordinal variable.
2. Application time was increased progressively as follows: 1-2-3-4-5 min. More than 5 min in alternating/pulsing mode. Classified as a continuous variable.
3. Suction was increased progressively to the maximum values. Classified as a continuous variable.
4. The vibration mode was a dichotomous variable: alternating/pulsing and continuous. Classified as a dichotomic variable.

The deterioration of intraoperative neurophysiological monitoring was considered the first outcome variable, considering the high sensitivity and specificity in predicting motor damage [17]. The second outcome variable was the assessment of the effect of corticosteroid medication on motor damage. The assessment of the effect of corticosteroid medications was performed as a qualitative analysis. A single bolus of betamethasone 8 mg was administered following a reduction of >50% or disappearance of motor potentials.

We excluded incomplete/missing data from the final analysis. For this reason, data retrieved from 2 experiments are not reported since it was not possible to fully complete the testing of the ultrasound aspirators due to anesthesiology-related issues.

### 2.5. Anesthesiologic Protocol

**Figure 1:**
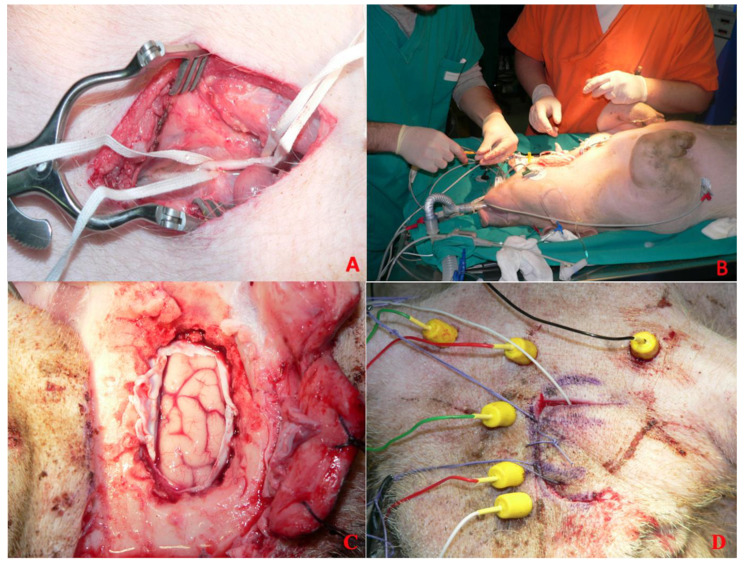
Anesthesiologic protocol. (**A**) Detail of the isolated internal carotid artery. (**B**) Procedure of cannulation of the internal carotid artery. (**C**) Cerebral cortex exposed for direct cortical stimulation after craniotomy and dural opening. (**D**) Corkscrew electrodes placement.

Induction was performed using propofol (6 mg/kg) and ketamine (10 mg/kg) via the marginal ear vein. The animals were intubated and mechanically ventilated with a mixture of air and oxygen (O_2_ 40%) at a rate of 10–12 breaths/min. Anesthesia and analgesia were maintained with continuous infusion of propofol (5 mg/kg/h) and fentanyl (0.20 μg/kg/min). Halogenated drugs were not used for the well-known effect on the neurophysiological monitoring [18]. The heart rate, respiratory rate, invasive blood pressure measurement through a probe positioned in the internal carotid artery, and oxygen saturation were monitored intraoperatively using standard techniques [19,20] (Figure 1A,B). Hydration was maintained using intravenous lactated Ringer’s solution. The temperature was measured by a rectal temperature probe and maintained at 35.0–37.0 °C.

At the end of the surgical procedure, the effective motor outcome of each animal was tested and, after electrophysiological data collection, the subjects were euthanized by injection of tanax (0.3 mL/kg) intravenously. The entire protocol was conducted and designed in collaboration between veterinarians and anesthesiologists.

### 2.6. Neurophysiological Protocol

Before the start of the surgical phase, the needle electrodes, twisted pair needle electrodes for the limbs, and corkscrew electrodes for the head were placed. The former were placed in proximity to the muscles of both fore and hind limbs (tibialis anterior and posterior), in the biceps brachii and quadriceps femoris (rectus femoris, vastus medialis, and vastus lateralis) of the 4 limbs [21]. The corkscrew electrodes, 6 in number, were in the skin of the head. For better recording, electrodes were placed in position Fpz, Fz, Cz, and Pz on the midline and C3 and C4, according to a slightly modified version of the 10–20 International system of Human EEG [22]. The reason for this modification compared to the standard system is due to the different anatomy of the swine.

The needles are connected to the NIM-ECLIPSE™ Spinal System apparatus. This equipment has 32 channels with which simultaneous electroencephalography (EEG), evoked potentials (EP), and electromyography (EMG) can be performed and detected. Pedicle monitoring with nerve stimulation, recording/detection of somatic and sensory response after stimulation, is also performed with this system. This system is routinely used in spinal surgery to have continuous neurophysiological monitoring.

Having arranged the needle electrodes and verified the correct positioning of the electrodes, motor evoked potentials (MEPs) and somato-sensory evoked potentials (SSEPs) are recorded (Figure 1C,D). This tracing will serve as a benchmark for potentials recorded later at regular intervals (5 min) and before and after each surgical phase. Direct cortical stimulation was performed to evaluate the response of each muscular group.

MEPs were evoked with trains of 5 impulses with a constant voltage range of 350–442 V. The duration of each pulse stimulation was 50 μs, with an interstimulus interval of 2 ms, and the current intensity of cortical stimulation ranged between 20 and 30 mA. Electroneurophysiological testing was performed by a clinical electrophysiologist with extensive experience in animal experimentation under the guidance of veterinarians to ensure correct execution of the protocol.

### 2.7. Surgical Protocol

**Figure 2:**
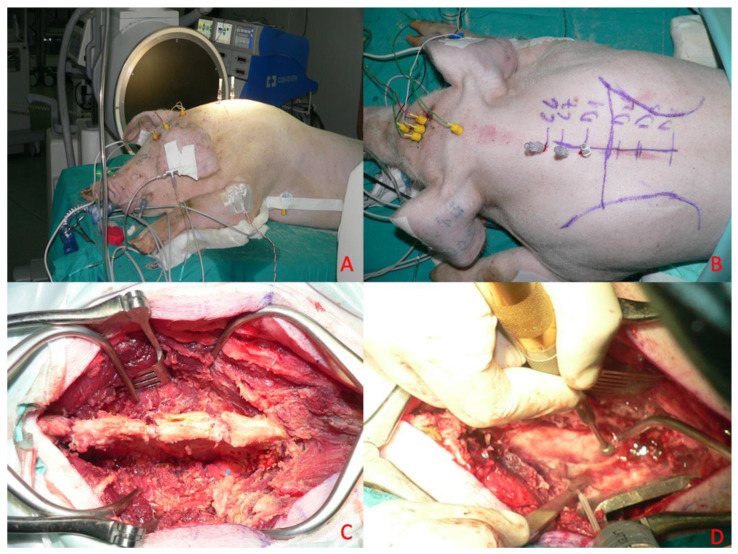
Surgical protocol. (**A**) Preoperative radioscopic inspection. (**B**) Skin incision. (**C**) Bilateral subperiosteal skeletonization. (**D**) D1-D3 laminectomies.

All subjects included in the present study were placed in the sternal position after general anesthesia and oro-tracheal intubation, and a neurophysiological monitoring system was set up. With the aid of an intraoperative radioscope (Siemens C-series—OES 9600), the C7-D4 spaces were identified, and on these spaces the skin was incised with a median longitudinal cut, and the subcutaneous tissue was peeled back until identification of the muscular planes (Figure 2).

Meanwhile, two 0.5 cm^3^ samples of porcine subcutaneous tissue were taken and stored in saline solution (NaCl 0.9%). Adipose tissue was chosen because it was easy to source, simple to divide into aliquots of known size, and reproducible in both sourcing and sizing. Most importantly, the consistency of the sample is similar to spinal cord tumors, resulting in suitable fragmentation and aspiration by both apparatuses under study. Using other material would not produce the desired effects, might be difficult to find (other surgeries in a different location), or may not have suitable characteristics for use. The D1-D3 laminae were skeletonized subperiosteally, and laminectomy was performed (Figure 2C). This was performed with the aid of a surgical drill and dedicated surgical instrumentation (Kerrison rongeurs) after subperiosteal disconnection of the paravertebral muscles and removal of the laminae (Figure 2D).

This surgical phase and subsequent phases were performed with a microsurgical technique employing an operating microscope with magnification up to 4×. At each stage, potentials were monitored before proceeding to the next stage.

We then proceeded with incision and suspension of the dura using 4.0 silk wires. Next, a pial suspension with Prolene 6.0 threads was performed. A median myelotomy was performed with a microscalpel, within which was placed the 0.5 cm^3^ sized autologous subcutaneous tissue fragment previously harvested and preserved in saline. In particular, the myelotomy in each procedure measured 1 cm; in each case, the size of the myelotomy was measured.

**Figure 3:**
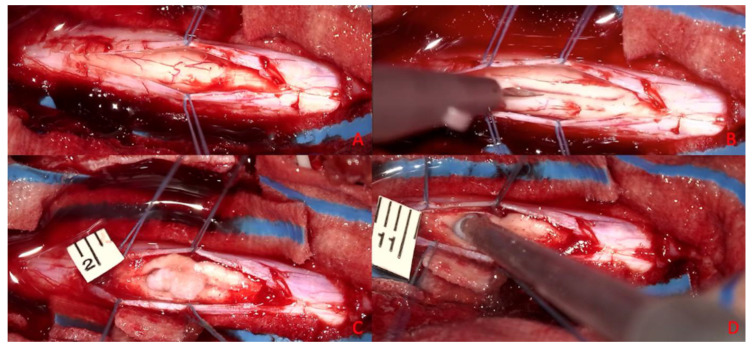
Microsurgical detail. (**A**) Dural opening. (**B**) Myelotomy. (**C**) Introduction of the target tissue. (**D**) Demolition of target tissue with the ultrasonic aspirator.

This fragment of subcutaneous tissue constitutes the surgical target of standard consistency and size useful for checking the damage transmitted to the perilesional nerve tissue during the use of the aspirator. After a 5 min waiting period, fragmentation and aspiration of the subcutaneous adipose tissue inserted into the spinal cord was performed using one of the two ultrasonic aspirators undergoing comparative evaluation (Figure 3).

Two samples of subcutaneous tissue were harvested, one for the testing of the continuous mode, the other for the pulsating mode. In half of the experiments, the pulsating/alternate mode was tested first and, in the other half, the continuous one was studied first to avoid biases related to the global time of testing.

Ultrasonic aspirators were never used directly on the medullary parenchyma but, after appropriate tests on the chosen phantom, were in contact only with the target sample.

The procedures were performed by the same surgeon and lasted about 4 h, during which (at set intervals of 5 min) and throughout the fragmentation and aspiration phase of the adipose tissue inserted into the medulla the neurophysiological response was recorded by monitoring changes in motor and sensory potentials concerning the phase of use of the ultrasonic aspirators during the removal of the autologous tissue induced in the spinal cord. Before the end of each procedure, a definitive decrease in the MEPs with neurophysiological findings was caused. (All major steps of the procedure are reported in Supplementary Video S1).

### 2.8. Sample Size

The study size was given by the initial experimental project. We addressed no missing data since incomplete records were the exclusion criteria. A potential source of bias is expected from the size of the sample, which nevertheless presents a fairly estimated power for a preliminary report.

### 2.9. Statistical Methods

The sample was analyzed using Python v3.13.0 to assess potential relationships between the variables under investigation. Initially, Chi-square (Chi^2^) tests were applied to evaluate associations between categorical variables, specifically assessing the impact of power (categorized as <30 Hz or >30 Hz), vibration mode (continuous or alternating/pulsatile), suction, and time (≤2 min or >2 min) on neurological impairment risks. Additionally, a two-way ANOVA test was conducted to compare mean values, particularly focusing on the effects of fragmentation power and time variables on outcomes. For continuous and ordinal variables, Pearson and Spearman correlations were used to investigate bivariate relationships and assess consistency in results across correlation methods. The threshold for statistical significance was set at *p* < 0.05.

## 3. Results

We excluded two experiments for aforementioned reasons. All 10 final experiments were carried out respecting the protocol, and motor damage due to the application of the ultrasound aspirator was registered in all cases. This precaution allowed the same results since no differences in the results obtained during the experiments were detected.

### 3.1. Primary Endpoints

**Figure 4:**
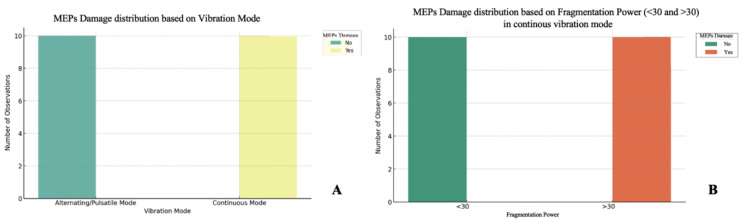
(**A**) Chi^2^ test. The vibration mode has a significant influence on MEPs decrease (*p* < 0.05). (**B**) Chi^2^ test. The power of fragmentation has a significant influence on MEPs decrease (*p* < 0.05) in continuous mode.

A decrease in MEPs occurs only under certain conditions, and analysis of the variables studied shows that the primary discriminant is the mode of vibration. Based on the findings during the 10 experiments with the same setting, it was seen that, regardless of application time, fragmentation power, and suction, in pulsed/alternating mode, no motor damage was obtained. In contrast, in continuous vibration mode, it is possible to have a decrease in MEPs, despite the instrument being kept exclusively on the tumor (Figure 4A).

Specifically, when analyzing the remaining variables individually, it was seen that, while the change in suction power does not have a significant impact on motor damage, the condition that leads to a decrease in MEPs is application with fragmentation power > 30 for a time > 2 min in continuous mode (Figure 4B).

**Figure 5:**
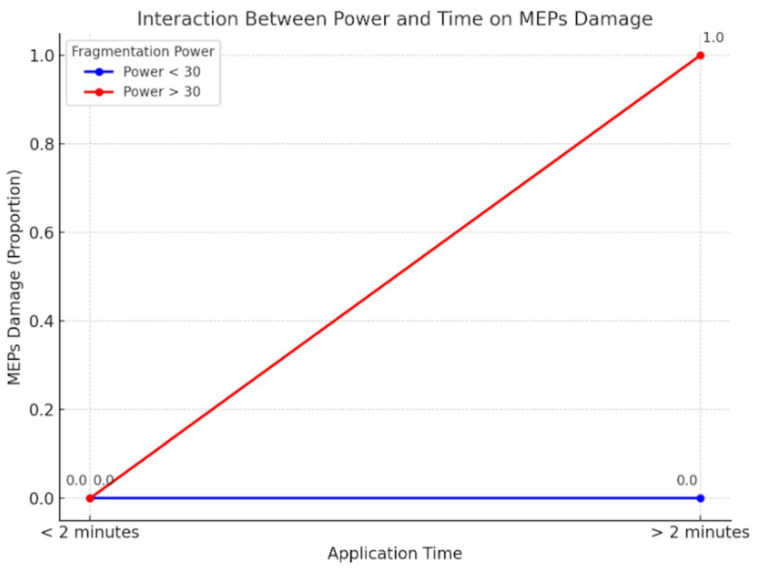
Two-way ANOVA test. The interaction between power and time is not statistically significant (*p*-value = 0.155), suggesting that the effect of time is strongly dependent on fragmentation power, but there is no significant interaction between the two.

At this point, a two-way ANOVA analysis was performed to test the interaction in continuous mode between application time and fragmentation power (Figure 5). The analysis showed the following results:

- Fragmentation power has a significant effect on MEP decrease (*p* < 0.001), confirming that there is a relationship between power and motor damage, with a cut-off of 30.
- Time is not statistically significant (*p* = 0.873), confirming that time alone does not cause a decrease in MEPs.
- The interaction between power and time is not statistically significant (*p* = 0.155), suggesting that the effect of time is strongly dependent on power, but there is no significant interaction between the two.

Therefore, according to these results, it is possible to assume that the most influential factor on motor damage is the power of fragmentation. All details regarding the possible scenarios are reported in Table 1.

### 3.2. Secondary Endpoint

**Figure 6:**
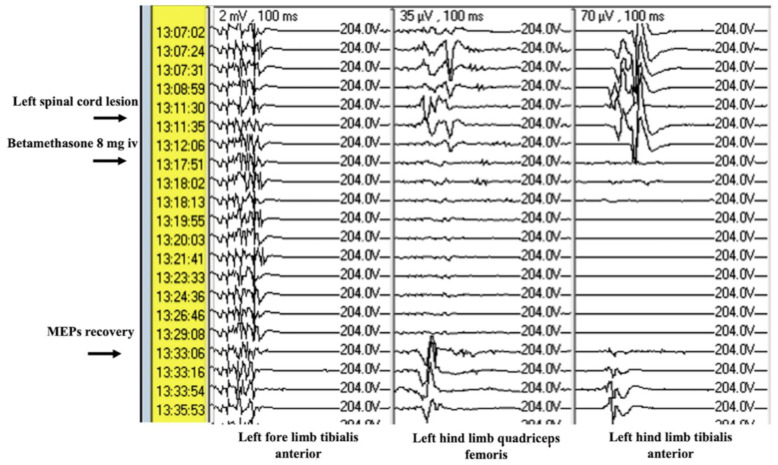
Upon the disappearance of potentials for the muscles innervating the gluteus and left lower limb, the procedure is immediately stopped, and a bolus of betamethasone 8 mg is administered. After 9 min, there is a reappearance of potentials in the absence of pathological changes.

Betamethasone was administered at a dosage of 8 mg immediately after injury, and MEPs showed recovery within a range of 8 to 17 min, with an average time of 12.5 min. This indicates a relatively rapid response and good efficacy of corticosteroid drugs in restoring neurophysiological signals (Figure 6).

## 4. Discussion

In the present study, we investigated the role of power, application time, suction vibration mode, and any interactions among these variables on MEPs impairment, attempting to delineate the critical factors contributing to spinal cord impairment when using the ultrasonic aspirator. The importance of intraoperative physiological monitoring in neurosurgery for the correct and safe performance of surgical procedures is a topic of crucial importance [17]. This aspect is particularly relevant in intramedullary tumor surgery, given the high risk of iatrogenic neurological damage inherent in surgical procedures. Another crucial aspect to consider is the medicolegal relevance of intraoperative neurophysiological monitoring (IONM) in neurosurgery. IONM not only helps prevent undetected electrophysiological changes during surgery but also provides objective documentation of neural functional integrity throughout the procedure. This documentation can be pivotal in legal contexts; numerous malpractice claims (approximately 54%) are related to the failure to implement IONM or its negligent use in cases where it was indicated [23].

The ultrasonic aspirator has been a widely used tool in neuro-oncology for decades, both for the treatment of intra- and extra-axial brain and spinal cord tumors [24]. Although the specific techniques used with these instruments are the same regardless of the encephalic or spinal location of the tumor, the possibility that, in specific situations, the ultrasonic aspirator may cause damage to healthy spinal cord tissue comes from clinical experiences, finding no clear data in the literature regarding the efficacy/safety profile. However, other authors in the literature have shown that, during surgery of intramedullary tumors, the use of the ultrasonic aspirator is linked to a decrease in MEPs, although none have focused on the specific settings of the instrument during surgery [8,9].

A first study of potential ultrasound damage on nerve tissue was carried out experimentally by Young et al. [10]. In this study, it was proved that, upon histological analysis of spinal cord and sciatic nerve on rat, it was possible to find damage from continuous use of the ultrasonic aspirator for an area immediately adjacent to the point of application; however, it is important to specify that the experiment involved application directly on healthy nerve tissue and not on pathological tissue.

In a small case series on the treatment of spinal tumors, both intra- and extramedullary, Suetsuna et al. [11] suggested that the use of ultrasound could cause a decrease in MEPs after even a few seconds at high power of application (>60), arguing that, at increasing values, the time of damage onset was shorter. In addition, since this was a generic study on spinal tumors, it was seen that prolonged use, even on the dura, may first cause swelling of the dura itself after prolonged use, leading to rupture with increasing power [11].

Barzilai et al. report a study on intramedullary tumors. They used ultrasonic aspirators with stimulation at the tip that allowed them to collect real-time data regarding the decrease in motor potentials [12].

It is evident that, over the years, the idea that one or more parameters related to the use of the ultrasonic aspirator in intramedullary tumor surgery may play a role in damage to healthy tissue.

The analysis demonstrated that the power of fragmentation is a critical factor in the occurrence of MEPs decrease. Our experiments, confirmed by the statistical analysis, revealed that exceeding the threshold of 30 is significantly associated with an increased risk of MEPs decrease when the ultrasound aspirator is used with the continuous vibration mode. Thus, the power of fragmentation appears to be the main parameter that can influence the probability of the impairment of neurophysiological signals, regardless of the duration of application. Observations indicated that, for power less than 30, a decrease in MEPs does not occur, even with prolonged application times, regardless of the vibration mode.

An interesting finding that emerged from the analyses concerns application time. Contrary to initial assumptions, application time did not prove to be a determining factor in the occurrence of MEP decrease, especially in continuous mode. Statistical analyses, including ANOVA, showed that time (≤2 min vs. >2 min) has no significant influence on the incidence of damage if power is kept below the critical threshold of 30. This suggests that, below certain frequency thresholds, exposure duration is not a relevant parameter for neurological safety.

The vibration mode factor proved to be of fundamental importance in understanding the mechanism of damage. In alternating/pulsed mode, a decrease in MEPs does not occur, regardless of power or time of application. However, in continuous mode, power plays a key role; with power above 30, the risk of a decrease in MEPs increases significantly. The continuous mode would seem to be less safe, as high power of fragmentation exerts more stress on the surrounding tissues, likely due to continuous vibrations that compromise the microcirculation and neuronal microenvironment. However, below a threshold of 30, continuous mode does not lead to harm, suggesting that the power of fragmentation management is crucial for prevention.

Corticosteroid drugs demonstrated clear efficacy in restoring MEPs after injury. Administered at a dosage of 8 mg immediately after the onset of reduction or disappearance of MEPs signals, betamethasone allowed recovery of signals within 8 to 17 min. This suggests that betamethasone, probably due to its anti-inflammatory action, may reduce inflammation-induced secondary damage and improve local blood flow. The results indicate that, during reversible neurophysiological damage, early intervention with corticosteroid drugs may prevent permanent damage to the spinal cord.

### Limitations and Future Perspectives

The main limitation of this study concerns the fact that, even if the swine model and the experiment’s setting aim to recreate a real surgical intervention, this protocol cannot completely reproduce the simulation of a spinal cord tumor in a human subject. Given the impossibility of performing experiments on humans for obvious reasons, other kinds of artificial simulations are needed to confirm the results of this study.

Another limitation of this study is the small sample size, which was appropriate for the reproducibility of the experimental model, but a larger group is needed for further validation of this preliminary data.

The choice of the subcutaneous fat tissue as the target for the experimental fact is widely explained in the manuscript. However, this model does not reproduce one of the features of intramedullary tumors: the adhesion to the normal spinal cord tissue. Even though this feature is not reproduced, the use of the ultrasound aspirator on the borders of the sample, directly adjacent to the normal spinal cord tissue, is a reliable reproduction of the effects of ultrasound on normal spinal cord.

Considering the data retrieved from our experiments, questions automatically arise, which first concern whether there is a way to safely use the ultrasound aspirators even in continuous mode and for high power of fragmentation in the proximity of the spinal cord. In addition, the question remains open as to identifying all the factors involved in causing spinal cord ischemia: the peculiar circulation of the spinal cord, the use of this instrument in a small-diameter channel surrounded by bone, other morphological and physiological parameters of the spinal cord, or the combination of these.

Finally, we analyzed the extent and type of histological damage caused by the ultrasonic aspirator. Although the results showed histological damage, the inability to obtain high-quality histological images led us to remove this analysis from the manuscript.

## 5. Conclusions

Based on our experimental experience with the present swine model, we can state that the discontinuous modes (pulsatile or alternating) provide safe and effective use of ultrasonic aspirators even for high fragmentation power and longer application times. Even in continuous mode, an ultrasonic aspirator can be used effectively, using it with lower fragmentation power to have a safety margin. More specifically, our experiments demonstrated that the ultrasound aspirator can cause a significant decrease in the MEP signal when used in continuous mode for more than 2 min, with fragmentation power >30. Application time alone does not affect the creation of any motor damage, nor does suction.

Immediate administration of boluses of 8 mg of betamethasone can reverse MEPs decrease, with an average effect time of 12.5 min.

These findings must be confirmed in future studies, in consideration of the limitations of the present model.

## Figures and Tables

**Table 1:** Motor outcome depending on the combination of vibration mode, power, and time of application.

Fragmentation Power	Modality	Time	MEPs Decrease (Yes/No)
<30	Any	Any	No
>30	Continous	>2 min	Yes
>30	Continous	<2 min	No
>30	Alternate/Pulsating	>2 min	No
>30	Alternate/Pulsating	<2 min	No

## Data Availability

Data available upon request to the corresponding author due to legal reasons.

## Supplementary Materials

The following supporting information can be downloaded at: https://www.mdpi.com/article/10.3390/brainsci15070670/s1, File S1: ARRIVE guidelines checklist; Video S1: showing all the major steps of one of the experiments. References [25,26] are cited in the supplementary materials.

## Abbreviations

The following abbreviations are used in this manuscript:

CNS	Central Nervous System
EEG	Electroencephalography
EM-SPT	Extramedullary intradural spinal tumors
EMG	Electromyography
EP	Evoked potentials
IM-SCT	Intramedullary spinal tumors
IONM	Intraoperative neuromonitoring
MEPs	Motor evoked potentials
SSEPs	Somato-sensory evoked potentials

## References

[1] Liu L., Shi L., Su Y., Wang K., Wang H. (2024). Epidemiological features of spinal intradural tumors, a single-center clinical study in Beijing, China. BMC Musculoskelet. Disord..

[2] Louis D.N., Perry A., Wesseling P., Brat D.J., Cree I.A., Figarella-Branger D., Hawkins C., Ng H.K., Pfister S.M., Reifenberger G. (2021). The 2021 WHO classification of tumors of the central nervous system: A summary. Neuro Oncol..

[3] Raco A., Piccirilli M., Landi A., Lenzi J., Delfini R., Cantore G. (2010). High-grade intramedullary astrocytomas: 30 years’ experience at the Neurosurgery Department of the University of Rome “Sapienza”. J. Neurosurg. Spine.

[4] Raco A., Esposito V., Lenzi J., Piccirilli M., Delfini R., Cantore G. (2005). Long-term follow-up of intramedullary spinal cord tumors: A series of 202 cases. Neurosurgery.

[5] Zurita Perea S.N., Alvarez Abut P.A., Seidel K. (2024). A Concise Guide to D-Wave Monitoring during Intramedullary Spinal Cord Tumour Surgery. Medicina.

[6] Witt H., Gramatzki D., Hentschel B., Pajtler K.W., Felsberg J., Schackert G., Löffler M., Capper D., Sahm F., Sill M. (2018). DNA methylation-based classification of ependymomas in adulthood: Implications for diagnosis and treatment. Neuro Oncol..

[7] Abd-El-Barr M.M., Huang K.T., Moses Z.B., Iorgulescu J.B., Chi J.H. (2018). Recent advances in intradural spinal tumors. Neuro Oncol..

[8] Cheng J.S., Ivan M.E., Stapleton C.J., Quinones-Hinojosa A., Gupta N., Auguste K.I. (2014). Intraoperative changes in transcranial motor evoked potentials and somatosensory evoked potentials predicting outcome in children with intramedullary spinal cord tumors. J. Neurosurg. Pediatr..

[9] Constantini S., Miller D.C., Allen J.C., Rorke L.B., Freed D., Epstein F.J. (2000). Radical excision of intramedullary spinal cord tumors: Surgical morbidity and long-term follow-up evaluation in 164 children and young adults. J. Neurosurg..

[10] Young W., Cohen A.R., Hunt C.D., Ransohoff J. (1981). Acute physiological effects of ultrasonic vibrations on nervous tissue. Neurosurgery.

[11] Suetsuna F., Harata S., Yoshimura N. (1991). Influence of the ultrasonic surgical aspirator on the dura and spinal cord. An electrohistologic study. Spine.

[12] Barzilai O., Lidar Z., Constantini S., Salame K., Bitan-Talmor Y., Korn A. (2017). Continuous mapping of the corticospinal tracts in intramedullary spinal cord tumor surgery using an electrified ultrasonic aspirator. J. Neurosurg. Spine.

[13] Gutierrez K., Dicks N., Glanzner W.G., Agellon L.B., Bordignon V. (2015). Efficacy of the porcine species in biomedical research. Front. Genet..

[14] Gavira N., Benayoun M., Hamel Q., Fournier H.D., Bigorre N. (2022). Learning, teaching, and training in microsurgery: A systematic review. Hand Surg. Rehabil..

[15] Walters E.M., Prather R.S. (2013). Advancing swine models for human health and diseases. Mo. Med..

[16] Percie du Sert N., Hurst V., Ahluwalia A., Alam S., Avey M.T., Baker M., Browne W.J., Clark A., Cuthill I.C., Dirnagl U. (2020). The ARRIVE guidelines 2.0: Updated guidelines for reporting animal research. Br. J. Pharmacol..

[17] Tropeano M.P., Rossini Z., Franzini A., Capo G., Olei S., De Robertis M., Milani D., Fornari M., Pessina F. (2023). Multimodal Intraoperative Neurophysiological Monitoring in Intramedullary Spinal Cord Tumors: A 10-Year Single Center Experience. Cancers.

[18] Xiang B., Jiao S., Zhang Y., Wang L., Yao Y., Yuan F., Chen R., Zhou Q. (2021). Effects of desflurane and sevoflurane on somatosensory-evoked and motor-evoked potential monitoring during neurosurgery: A randomized controlled trial. BMC Anesthesiol..

[19] Montes E., Burgos J., Barrios C., de Blas G., Hevia E., Forteza J. (2017). Neurophysiological monitoring during acute and progressive experimentally induced compression injury of the spinal cord in pigs. Eur. Spine J..

[20] Hu C.K., Chen M.H., Wang Y.H., Sun J.S., Wu C.Y. (2023). Integration of multiple prognostic predictors in a porcine spinal cord injury model: A further step closer to reality. Front. Neurol..

[21] Mok J.M., Lyon R., Lieberman J.A., Cloyd J.M., Burch S. (2008). Monitoring of nerve root injury using transcranial motor-evoked potentials in a pig model. Spine.

[22] Klem G.H., Lüders H.O., Jasper H.H., Elger C. (1999). The ten-twenty electrode system of the International Federation. The International Federation of Clinical Neurophysiology. Electroencephalogr. Clin. Neurophysiol. Suppl..

[23] Hatef J., Katzir M., Toop N., Islam M., Clark T., Roscoe C., Khan S., Mendel E. (2020). Damned if you monitor, damned if you don’t: Medical malpractice and intraoperative neuromonitoring for spinal surgery. Neurosurg. Focus..

[24] Ahmad N., Bakhshi S.K., Shamim M.S. (2021). Use of ultrasonic aspirator for CNS tumour resection. J. Pak. Med. Assoc..

[25] Kilkenny C., Browne W.J., Cuthill I.C., Emerson M., Altman D.G. (2010). Improving Bioscience Research Reporting: The ARRIVE Guidelines for Reporting Animal Research. PLoS Biol..

[26] Schulz K.F., Altman D.G., Moher D., the CONSORT Group (2010). CONSORT 2010 Statement: Updated guidelines for reporting parallel group randomised trials. BMJ.

